# Virus Infection Triggers MAVS Polymers of Distinct Molecular Weight

**DOI:** 10.3390/v10020056

**Published:** 2018-01-30

**Authors:** Natalia Zamorano Cuervo, Quentin Osseman, Nathalie Grandvaux

**Affiliations:** 1 CRCHUM—Centre Hospitalier de l’Université de Montréal, 900 rue Saint Denis, Montréal, QC H2X 0A9, Canada; Natalia.zamorano@umontreal.ca (N.Z.C.); qosseman@gmail.com (Q.O.); 2 Department of Biochemistry and Molecular Medicine, Faculty of Medicine, Université de Montréal, Montréal, QC H3C 3J7, Canada

**Keywords:** mitochondrial antiviral signaling (MAVS), virus, antiviral, interferon, pathogen recognition receptors (PRRs), oligomerization, aggregation

## Abstract

The mitochondrial antiviral signaling (MAVS) adaptor protein is a central signaling hub required for cells to mount an antiviral response following virus sensing by retinoic acid-inducible gene I (RIG-I)-like receptors. MAVS localizes in the membrane of mitochondria and peroxisomes and in mitochondrial-associated endoplasmic reticulum membranes. Structural and functional studies have revealed that MAVS activity relies on the formation of functional high molecular weight prion-like aggregates. The formation of protein aggregates typically relies on a dynamic transition between oligomerization and aggregation states. The existence of intermediate state(s) of MAVS polymers, other than aggregates, has not yet been documented. Here, we used a combination of non-reducing SDS-PAGE and semi-denaturing detergent agarose gel electrophoresis (SDD-AGE) to resolve whole cell extract preparations to distinguish MAVS polymerization states. While SDD-AGE analysis of whole cell extracts revealed the formation of previously described high molecular weight prion-like aggregates upon constitutively active RIG-I ectopic expression and virus infection, non-reducing SDS-PAGE allowed us to demonstrate the induction of lower molecular weight oligomers. Cleavage of MAVS using the NS3/4A protease revealed that anchoring to intracellular membranes is required for the appropriate polymerization into active high molecular weight aggregates. Altogether, our data suggest that RIG-I-dependent MAVS activation involves the coexistence of MAVS polymers with distinct molecular weights.

## 1. Introduction

Cells respond to invading viruses through the activation of specific signaling cascades that ultimately elicit the induction of an antiviral transcriptional program. The early events of this response involve viral nucleic acid sensing by multiple pathogen recognition receptors (PRRs). Amongst the PRRs, the retinoic acid-inducible gene I (RIG-I)-like receptors (RLRs), RIG-I, and melanoma differentiation-associated gene 5 (MDA5) [1,2], sense viral RNA replication intermediates in the cytosol [3,4]. The binding of RNA moieties to RIG-I/MDA5 induces an interaction with the mitochondrial antiviral signaling (MAVS) adaptor, also known as interferon beta promoter stimulator 1 (IPS-1), caspase activation recruitment domain (CARD) adaptor inducing interferon beta (CARDIF), or virus-induced signaling adaptor (VISA) [5,6,7,8]. Initial characterization of MAVS reported its localization in the outer membrane of mitochondria [5] but subsequent research has demonstrated additional localization in the membrane of peroxisomes [9] and in mitochondrial-associated endoplasmic reticulum membranes (MAMs) [10]. The MAVS adaptor serves as a key signaling hub that constitutes an interface for the direct binding of various signaling proteins, including the tumor necrosis factor (TNF) receptor-associated factor (TRAF) 3 and TRAF6 adaptors, thereby forming a macromolecular signaling complex known as the signalosome [11,12,13]. These interactions trigger multiple downstream signaling pathways that ultimately lead to the coordinated activation of the interferon (IFN) regulatory factors (IRFs) and nuclear factor-κB (NF-κB) transcription factors [14,15] to regulate the expression of type I and III IFNs, proinflammatory cytokines, and a subset of IFN-stimulated genes (ISGs) [3,4,16]. Attempts to determine the role of MAVS in gene regulation using constructs encoding fusion with organelle-targeting signals or peroxisome-deficient cells has led to inconsistent observations, leaving the selective role of mitochondrial vs. peroxisomal MAVS unclear [9,17,18,19].

MAVS is a transmembrane protein with a long cytoplasmic domain, a short intermembrane/luminal domain, and a transmembrane domain organized in α-helices. The N-terminal (CARD) domain in the cytoplasmic region is essential for MAVS activity [5]. Upon binding of viral RNA, RIG-I undergoes a conformational change. This leads to the exposure of two N-terminal CARD domains that become involved in a CARD-CARD interaction with MAVS [20,21], initiating the conversion of MAVS monomers into functional self-perpetuating protease- and detergent-resistant aggregates exhibiting α-helix structures [22,23]. The analyses of purified recombinant MAVS protein by crystallography, electron microscopy, or by solid-state nuclear magnetic resonance (NMR) have been successful for deciphering the α-helix structure and the molecular requirements for the initiation of MAVS aggregation [20,21,23,24,25,26]. Using size-exclusion chromatography (SEC), the group of J. Chen at the University of Texas Southwestern Medical Center was able to separate recombinant MAVS lacking the transmembrane domain into two fractions of low and high molecular weight (MW) [22]. Using an in vitro IRF3 dimerization assay, they showed that only the high MW fraction was capable of activating IRF3 [22]. Several tools and techniques have been developed to assess MAVS dimerization and aggregation in a cellular context. First, MAVS self-association was observed using fluorescence resonance energy transfer (FRET) and co-immunoprecipitation assays [27]. However, these techniques are limited to ectopically expressed MAVS and do not allow detection of polymer formation and size characterization. Sendai virus (SeV)-induced MAVS aggregates were observed through the resolution of purified crude mitochondria extracts by semi-denaturing detergent agarose gel electrophoresis (SDD-AGE) [22]. Since then, the combination of purified mitochondria extraction and SDD-AGE has been the gold standard technique used to monitor mitochondria-associated MAVS aggregation in a cellular context [23,28,29].

Protein aggregation is a dynamic process initiated by a nucleation step that leads to monomer assembly into homo- or hetero-oligomers. Elongation of the newly formed low MW oligomers leads to protofibril intermediates that ultimately become high MW aggregates [30,31,32,33]. In contrast to aggregates, oligomer species are heterogeneous and are thought to underlie the etiology of aggregate-associated diseases [32]. Therefore, characterization of the oligomeric intermediates is crucial for understanding the dynamics of the aggregation process. The formation of MAVS oligomers has not yet been documented. Here, using a single-step whole cell extract (WCE) preparation coupled to non-reducing SDS-PAGE and SDD-AGE analyses, we report the coexistence of MAVS oligomers and high MW aggregates upon constitutively active RIG-I ectopic expression and virus infection. Anchoring of MAVS to intracellular membranes appears to be essential for an appropriate polymerization process allowing functional high MW aggregates to occur.

## 2. Materials and Methods

### 2.1. Plasmids

The pcDNA3.1 Hygro-Myc-MAVS plasmid and the pCDNA3.1 Zeo-Myc-ΔRIGI plasmid encoding the N-terminal CARD domains of RIG-I (aa 1–280) [34]) were obtained from Dr. Rongtuan Lin, McGill University, Montreal, Canada. The HR’TRIPΔU3 (CMV)-NS3/4A and the HR’TRIPΔU3 (CMV)-GFP plasmids were described previously [34].

### 2.2. Cell Culture

A549 and human embryonic kidney (HEK) 293 cells (American Type Culture Collection, ATCC, Manassas, VA, USA) were grown in F12 and DMEM medium (GIBCO, Life Technologies Inc., Burlington, ON, Canada), respectively. Media were supplemented with 1% l-glutamine and 10% FetalClone III (HyClone, Logan, UT, USA). All cultures were performed without antibiotics and were regularly controlled for mycoplasma contamination using the MycoAlert Mycoplasma Detection Kit (Lonza, Basel, Switzerland).

### 2.3. Plasmid Transfection

Plasmid transfection in A549 cells was achieved using the TransIT-LT1 transfection reagent (Mirus, Madison, WI, USA). Briefly, a total of 6 μg of DNA was transfected per 60 mm plate of A549 cells at 70% confluency using a TransIT-LT1/DNA ratio of 1:2. HEK293 cells were transfected with a total of 5 μg of DNA per 35 mm plate using the calcium phosphate co-precipitation method. Transfection was pursued 24 h before further treatment.

### 2.4. Virus Infection

Sendai virus (SeV) Cantell strain (Charles River Laboratories, Wilmington, MA, USA) was used to infect subconfluent A549 cells in 60 mm plates. Sixteen hours before infection, the culture medium was replaced by DMEM without phenol red and supplemented with 1% l-glutamine and 2% FetalClone III. Infection was performed by adding SeV at 10 HAU/10^6^ cells in serum-free medium (SFM). At 2 h post-infection, 2% FetalClone III was added and the infection was pursued for the indicated time.

### 2.5. Cell Lysis

All steps were performed on ice. Cells were scraped in cold PBS completed with 100 mM *N*-ethylmaleimide (NEM, Sigma Aldrich, Saint Louis, MO, USA), transferred into pre-chilled 1.5 mL tubes and pelleted by centrifugation at 16,200× *g* for 20 s at 4 °C. Pellets were immediately resuspended in 70 μL cold PBS supplemented with 100 mM NEM, 10 µg/mL aprotinin, 10 µg/mL leupeptin, 5 mM NaF, 1 mM activated Na_3_VO_4_, 2 mM *p*-nitrophenyl phosphate, and 10 mM β-glycerophosphate (Buffer I). Samples were sonicated using 3 × 15 pulses of 0.8 s at 40% (UP50H sonicator, Hielscher) with rounds on ice in between. After centrifugation at 800× *g* for 10 min at 4 °C, the supernatants were transferred into a clean pre-chilled 1.5 mL tube before adding an equal volume of cold Buffer I supplemented with 0.1% SDS. Samples were incubated on ice for 15 min and quantified using the BCA method (Pierce BCA Protein Assay Kit, Thermo Fisher Scientific, Waltham, MA, USA). Freezing of samples was found to be detrimental to the detection of aggregates; therefore, WCEs were analyzed immediately by SDD-AGE or non-reducing SDS-PAGE as described below.

### 2.6. SDD-AGE

WCEs were analyzed by SDD-AGE according to the protocol detailed in [35] with the modifications reported in [22] to analyze MAVS. Fresh WCEs (15 μg), prepared as detailed above, were completed with one-third volume of 4× loading dye consisting of 20% glycerol, 8% SDS, and bromophenol blue in 2× TAE buffer. Samples of less than 15 µL were completed to 15 µL with 1× loading dye. Samples were incubated for 5 min at room temperature. Prepared WCEs were resolved on a vertical 1.5% agarose gel (10 cm height; gel thickness 1.5 mm; 15 wells) in 1× TAE containing a final concentration of 0.1% SDS. Samples were gently loaded with long loading tips to have a minimum of sample height in the well to ensure that aggregate migration would be as homogeneous as possible. Migration was performed at 100 V for 35 min at 4 °C. An ice cube was added to the migration tank to maintain a low temperature and avoid sample diffusion. Proteins were transferred onto a nitrocellulose membrane using liquid transfer carried out overnight at 4 °C at 20 V constant in a transfer buffer containing 25 mM Tris base, 192 mM glycine, 0.1% SDS, and 10% EtOH. Prior to incubation with primary antibodies as described in the immunoblot section below, membranes were treated with 0.5% glutaraldehyde (LifeSensors, Malvern, PA, USA) in PBS for 20 min at room temperature followed by three washes in PBS [36].

### 2.7. Non-Reducing/Reducing SDS-PAGE

SDS-PAGE gels were prepared as detailed in [37] with a separation gel of 12% acrylamide/*bis*-acrylamide (37.5:1) and a stacking buffer of 3% acrylamide/*bis*-acrylamide (37.5:1). Fifteen micrograms of freshly prepared WCEs, as described above, were mixed with one-fourth volume of 5× loading buffer composed of 125 mM Tris HCl pH 6.8 (RT), 10% SDS, 20% glycerol, 0.0005% bromophenol blue with (reducing conditions) or without (non-reducing conditions) 3.6 M β-mercaptoethanol (BME). Samples were heated at 100 °C for 5 min and heating was briefly quenched on ice. Samples were immediately loaded on gels using long loading tips to achieve the lowest sample height possible for best resolution. Importantly, samples without BME were loaded away from samples containing BME. Migration was performed as described in [37]. Proteins in the stacking and resolution gels were transferred onto nitrocellulose membranes as detailed in [37] using transfer buffer supplemented with 20% ethanol and 0.036% SDS. Importantly, samples that were compared in reducing- and non-reducing-SDS PAGE were resolved on the same gel and presented in figures with the same immunoblot exposure.

### 2.8. Immunoblot

Nitrocellulose membranes were incubated with the following primary antibodies: anti-actin (Cat #A5441 from Sigma), anti-IFIT1 (Cat #14769S from Cell signaling, Danvers, MA, USA), anti-MAVS (Cat #A300-782AM from Bethyl, Montgomery, TX, USA), anti-Myc (Cat #M4439 from Sigma), and anti-NS3 (Cat #1847 from Virostat, Westbrook, ME, USA). After washes in PBS containing 0.5% Tween, membranes were further incubated with horseradish peroxidase (HRP)-conjugated secondary antibodies (KPL, Gaithersburg, MD, USA or Jackson Immunoresearch Laboratories, West Grove, PA, USA). Antibodies were diluted in PBS containing 0.5% Tween and either 5% nonfat dry milk or BSA. Immunoreactive bands were visualized by enhanced chemiluminescence using Western Lightning Chemiluminescence Reagent Plus (Perkin-Elmer Life Sciences, Waltham, MA, USA). Chemiluminescence signal acquisition was performed using an LAS4000mini CCD camera apparatus (GE healthcare, Little Chalfant, UK).

## 3. Results

### 3.1. WCE Analysis by SDD-AGE and Non-Reducing SDS-PAGE Reveals MAVS Aggregates and Oligomers of Distinct MW Induced by SeV Infection

It is well established that virus infections sensed by the RLRs pathway trigger MAVS aggregation and activation [22,23]. However, aggregates only represent the ultimate stage of a dynamic process that involves oligomeric intermediate states. Oligomers play an important role in the formation of aggregates and they are sometimes considered as an obligatory intermediate state between the monomer and the final aggregate [38]. Therefore, we sought to evaluate whether virus infection induces multiple polymerization states of MAVS. Because of their high MW, techniques such as SDD-AGE have been valuable for identifying MAVS aggregates [35]. In SDD-AGE, the use of agarose-based gels for protein resolution favors the migration of high MW fibrils. Oligomers exhibit lower MW that is compatible with resolution by acrylamide gel-based electrophoresis. Thus, we exploited these differences and used agarose and acrylamide gel-based electrophoresis to distinguish MAVS polymer species. Since previous reports have shown that MAVS aggregates are sensitive to treatment with BME [22], we assessed MAVS forms by SDS-PAGE in non-reducing conditions using the same WCEs analyzed by reducing SDS-PAGE and SDD-AGE.

**Figure 1:**
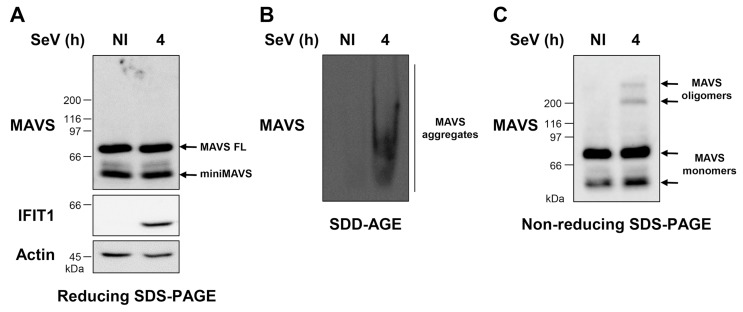
Detection of endogenous mitochondrial antiviral signaling (MAVS) protein aggregates and oligomers induced upon Sendai virus (SeV) infection. (**A**–**C**) A549 cells were infected with SeV at 10 HAU/10^6^ cells for 4 h. Preparation of whole cell extract (WCE) was performed immediately after harvesting. In (**A**) and (**C**), WCEs were subjected to SDS-PAGE after denaturation in SDS loading buffer with (**A**) or without (**C**) BME. Proteins were detected by immunoblot using anti-MAVS, anti-IFIT1, and anti-actin antibodies. In (**B**), WCEs were resolved by semi-denaturing detergent agarose gel electrophoresis (SDD-AGE) and analyzed by immunoblot using anti-MAVS antibodies.

First, to determine whether virus infection triggers diverse species of MAVS polymers (i.e., oligomer intermediates and high MW aggregates), A549 cells were infected with SeV at 10 HAU/10^6^ cells for 4 h. Cells were harvested and processed immediately. WCEs were prepared as detailed in Materials and Methods and the same WCE was simultaneously subjected to resolution by SDD-AGE and reducing- and non-reducing SDS-PAGE. Detection of MAVS by immunoblot after reducing SDS-PAGE revealed the two typical forms of MAVS: the full-length MAVS (MAVS FL) at 72 kDa MW and the truncated form, also referred to as miniMAVS, at around 50 kDa [39] (Figure 1A). Detection of IFN-induced proteins with tetratricopeptide repeats 1 (IFIT1) by immunoblot confirmed the efficient induction of an antiviral response during the timeframe of the infection (Figure 1A).

As previously documented, SeV infection induced the formation of endogenous MAVS high MW aggregates, which are detected by immunoblot following resolution by SDD-AGE (Figure 1B). Non-reducing SDS-PAGE electrophoresis analysis revealed that in non-infected cells, MAVS is detected at the expected apparent MW corresponding to monomeric forms of MAVS FL and miniMAVS (Figure 1C). While a substantial fraction of MAVS remains as monomers following SeV infection, MAVS is also detected as oligomeric species migrating with an apparent MW of 200–300 kDa (Figure 1C). Altogether, these results suggest that virus infection triggers the formation of the known described prion-like aggregates, as well as previously uncharacterized low MW oligomers, which can be detected independently through the analysis of total WCEs by a combination of SDD-AGE and non-reducing SDS-PAGE electrophoresis.

### 3.2. Activation of RIG-I Is Sufficient to Trigger MAVS Oligomerization and Aggregation

**Figure 2:**
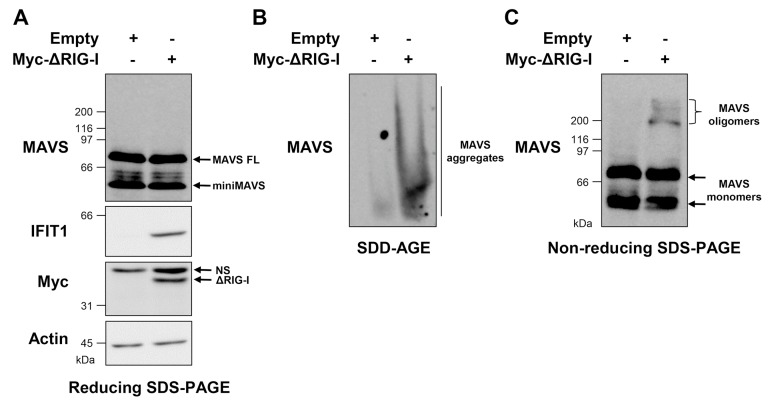
Activation of retinoic acid-inducible gene I (RIG-I) is sufficient to induce MAVS aggregation and oligomerization. A549 cells were transfected for 24 h with a plasmid encoding constitutively active RIG-I (Myc-ΔRIG-I) or an empty vector. The WCEs were prepared immediately after harvesting. WCEs were resolved by SDS-PAGE in reducing (**A**) or non-reducing (**C**) conditions followed by immunoblot analysis using anti-MAVS, anti-IFIT1, anti-Myc and anti-actin antibodies. In (**B**), WCEs were analyzed by SDD-AGE coupled to anti-MAVS immunoblot. NS: non-specific.

According to in vitro studies, MAVS aggregation is initiated by the interaction between the CARD domains of RIG-I and MAVS [20,21,22]. To determine whether RIG-I activation is sufficient to induce the formation of MAVS oligomers and aggregates in a cellular context, A549 cells were transfected with an empty plasmid or a plasmid encoding a constitutively active form of RIG-I (ΔRIG-I) [34]. The cells were harvested 24 h after transfection and lysed as described in Figure 1; WCEs were immediately subjected to SDD-AGE and non-reducing and reducing SDS-PAGE. Analysis by SDS-PAGE in standard reducing conditions revealed that expression of ΔRIG-I yields no change in the levels or profile of MAVS FL or miniMAVS, but leads to the induction of IFIT1, indicative of the activation of the antiviral response (Figure 2A).

The expression of ΔRIG-I was also found sufficient to induce the formation of high MW MAVS aggregates detected by the resolution of WCEs by SDD-AGE (Figure 2B). Analysis by SDS-PAGE in non-reducing conditions showed that while the major fraction of MAVS remains as monomers upon ΔRIG-I expression, MAVS also forms oligomeric species that migrate at an apparent MW of around 200–300 kDa (Figure 2C). Taken together, these results indicate that in a cellular context, activation of RIG-I is sufficient to induce the coexistence of MAVS low MW oligomers and high MW aggregates.

### 3.3. Ectopically Expressed MAVS Is Detected as Oligomeric and Aggregated States

**Figure 3:**
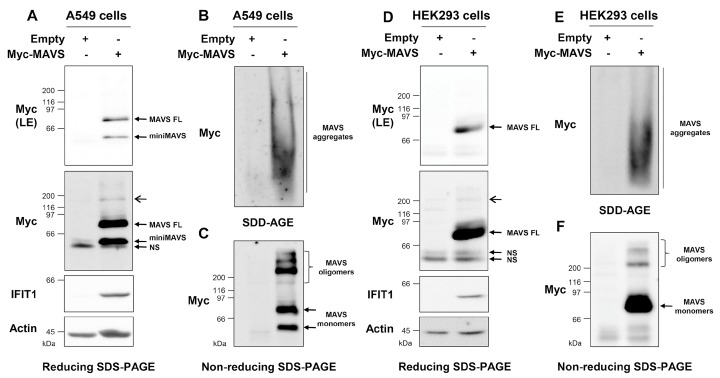
Detection of the aggregation and oligomerization states of ectopically expressed MAVS. A549 (**A**–**C**) and HEK293 (**D**–**F**) cells were transfected with a plasmid encoding for Myc-tagged MAVS (Myc-MAVS) or an empty vector. Cells were harvested, lysed, and WCEs were immediately subjected to reducing SDS-PAGE (**A**,**D**); SDD-AGE (**B**,**E**) or non-reducing SDS-PAGE (**C**,**F**); Proteins were detected by immunoblot using anti-actin, anti-Myc, or anti-IFIT1 antibodies. In (**A**,**D**), in addition to the exposure of the anti-Myc immunoblot corresponding to the exposure of panel (**C**,**F**), respectively, a lower exposure (LE) is shown for appropriate appreciation of miniMAVS. Open arrows indicate reducing-resistant forms of MAVS migrating at a MW higher than the monomers. NS: non-specific.

Ectopically expressed MAVS is constitutively active and FRET and co-immunoprecipitation assays were previously used to demonstrate that MAVS is present in cells as a polymer of at least two molecules [27,28]. We sought to use SDD-AGE and non-reducing SDS-PAGE to assess the state of oligomerization of constitutively active MAVS. A549 and HEK293 cells were transfected with an empty plasmid or a Myc-tagged MAVS-encoding plasmid. Cells were harvested 24 h post-transfection, lysed, and immediately processed through SDD-AGE and reducing and non-reducing SDS-PAGE. Immunoblot detection after standard reducing SDS-PAGE electrophoresis confirmed expression of Myc-MAVS and constitutive activation. Indeed, expression of Myc-MAVS was sufficient to induce IFIT1 expression in both cell types (Figure 3A,D). It is noteworthy that in A549 cells, Myc-MAVS is expressed as both MAVS FL and miniMAVS (Figure 3A), while in HEK293 the miniMAVS form is barely detectable (Figure 3D). Additionally, Myc-MAVS is consistently detected as upper MW reduction-resistant forms in both A549 and HEK293 cells (Figure 3A,D).

Analysis of WCEs by SDD-AGE followed by detection with anti-Myc antibodies demonstrates that constitutively active MAVS forms high MW aggregates in A549 and HEK293 cells (Figure 3B,E). Resolution of the same WCEs by non-reducing SDS-PAGE reveals a ladder profile corresponding to at least four distinct oligomers of increasing apparent MW, between 120 and 300 kDa (Figure 3C,F). Similar to what was observed for endogenous MAVS, a substantial fraction of Myc-MAVS migrates at a MW corresponding to the monomers (Figure 3A,D). These observations demonstrate that ectopically expressed constitutively active MAVS coexists as distinct MW polymers that include high MW aggregates and low MW oligomers.

### 3.4. MAVS Anchoring to Intracellular Membranes Is Essential for Functional Polymer Formation

**Figure 4:**
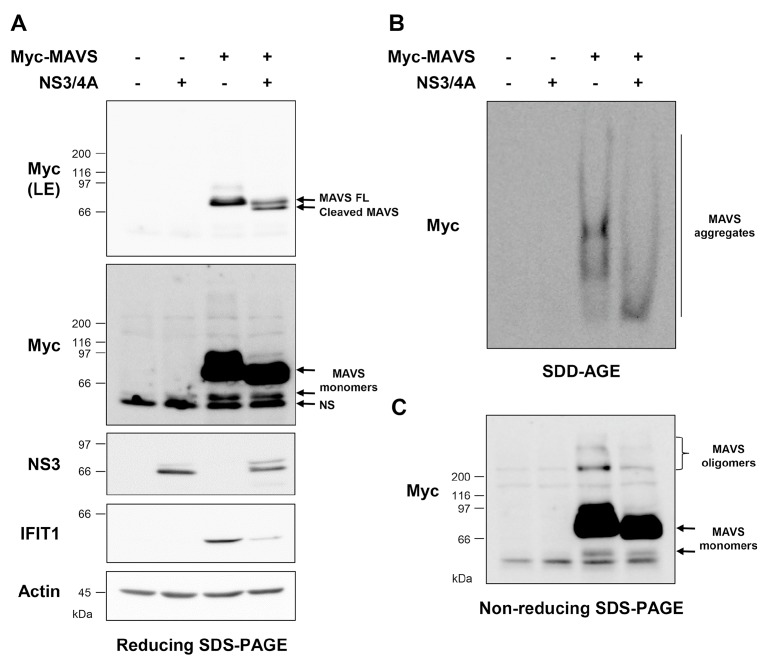
Impact of NS3/4A-mediated cleavage on the formation of MAVS polymer species. HEK293 cells were co-transfected with plasmids encoding NS3/4A and Myc-tagged MAVS (Myc-MAVS) or the corresponding empty vectors. At 24 h post-transfection, cells were harvested, lysed, and the WCEs were immediately subjected to reducing SDS-PAGE (**A**); SDD-AGE (**B**) or non-reducing SDS-PAGE (**C**). Proteins were detected by immunoblot using anti-actin, anti-Myc, anti-IFIT1, or anti-NS3 antibodies. In (**A**), in addition to the exposure of the anti-Myc immunoblot corresponding to the exposure of panel (**C**), a lower exposure is shown for appropriate appreciation of the MAVS cleavage by NS3/4A. NS: non-specific.

Previous reports have shown that MAVS anchoring to intracellular membranes is essential for its activation [9,18,40]. The Hepatitis C virus (HCV)-encoded NS3/4A protease inhibits the IFN antiviral response through cleavage of MAVS at the cysteine 508 upstream of the transmembrane domain, resulting in MAVS inactivation through relocalization to the cytoplasm [9,18]. To determine which MAVS polymer species formation is dependent on membrane anchoring, HEK293 cells were cotransfected with NS3/4A- and MAVS-encoding plasmids. Cells were harvested and processed 24 h post-transfection for immediate analysis through SDS-PAGE and SDD-AGE. Reducing SDS-PAGE confirmed MAVS expression and cleavage when coexpressed with NS3/4A (Figure 4A). As previously reported, cleavage of MAVS resulted in a marked decrease in the induction of IFIT1 reflecting the loss of MAVS activity (Figure 4A).

Analysis of the same WCEs by SDD-AGE showed that MAVS cleaved by NS3/4A is still capable of forming aggregates, although of a lower MW than the ones formed by uncleaved, membrane-anchored MAVS (Figure 4B). Analysis by non-reducing SDS-PAGE revealed that the formation of low MW oligomer species is highly impaired upon NS3/4A-mediated cleavage of MAVS (Figure 4C). Altogether, these findings point to the requirement of MAVS anchoring to intracellular membranes for MAVS polymerization. Importantly, while cytoplasmic MAVS is still able to form high MW aggregates, these species are not active given the abolishment of antiviral gene induction.

## 4. Discussion

MAVS is a central signaling hub that coordinates the activation of the antiviral response engaged following recognition of viral RNA replication intermediates by the RIG-I-like receptors in the cytoplasm of infected cells [3,4]. Current knowledge of the mechanisms underlying MAVS activation is limited to the observation that the active MAVS fraction anchored in the outer membrane of mitochondria forms self-perpetuating aggregates upon binding to RIG-I through CARD-CARD interaction [20,21]. The dynamics of MAVS polymerization are barely documented. In the present study, we report the detection of distinct MW polymers induced in response to SeV infection or RIG-I activation. Particularly, we demonstrate the coexistence of high MW aggregates with oligomeric intermediates, which determine the outcome of the polymerization process.

High MW aggregates and low MW oligomers were distinguished using two methods: SDD-AGE and non-reducing SDS-PAGE. Both techniques were used to resolve the same WCE. The detection of MAVS aggregates by SDD-AGE analysis of purified mitochondrial extracts was previously proven to be a highly valuable assay that led to the first demonstration of the existence of MAVS aggregates [22]. While resolution of purified mitochondrial extracts by SDD-AGE provides a very useful tool for monitoring MAVS activation in a cellular context, this experimental design cannot yield information about MAVS at locations other than mitochondria, such as at peroxisomes and MAMs [9,10], nor does it provide clues about the existence of subsets of oligomers with distinct sizes. Here, the use of a simple single-step WCE preparation coupled to SDD-AGE preserved the detection of MAVS high MW aggregates induced in response to RIG-I sensing of virus infection (Figure 1B, Figure 2B and Figure 3B,E). Importantly, resolution of the same WCEs by non-reducing SDS-PAGE also permitted the discrimination of oligomeric species. In conclusion, the experimental approach used in this study renders detection of high MW aggregates and low MW oligomers amenable to MAVS at all possible subcellular localizations. This approach provides novel experimental opportunities to study the impact of MAVS activators and inhibitors on the dynamic process leading to MAVS functional aggregation.

MAVS oligomers, induced by SeV infection (Figure 1C) or following ectopic expression of constitutively active RIG-I (Figure 2C), that coexist with high MW aggregates have an apparent MW of 200–300 kDa. MAVS complexes with a similar apparent MW have previously been described in HEK293 cells through analytical SEC of purified mitochondrial extracts [41]. However, these complexes, as well as high MW complexes of above 600 kDa, were observed in resting conditions. These results differ from our study and those of others as reduction-sensitive oligomers and high MW aggregates were only observed following MAVS activation [22,42]. Whether polymer species detected by reducing-SDS-PAGE and SDD-AGE and through SEC-based separation of MAVS complexes are identical will have to be addressed in future studies. Of note, in the reducing SDS-PAGE conditions, ectopically expressed MAVS, but not endogenous MAVS, migrates not only at the expected size for monomers but also as forms of higher apparent MW (Figure 3A,D) despite appropriate conditions of denaturation (i.e., 3.6 M BME coupled to heating at 100 °C [43,44]). Importantly, these same highly migrating forms were also detected in WCEs from cells transfected with flag-tagged MAVS, thereby excluding an artifact due to the Myc-tagged MAVS protein analyzed in this study. At this point, it is, however, difficult to exclude that these forms might result from MAVS overexpression or from partial aggregation caused by SDS as previously documented for membrane proteins [45]. However, resolution of the same WCEs by non-reducing SDS-PAGE clearly reveals a different profile of migration, demonstrating the existence of distinct reduction-sensitive MAVS oligomers (Figure 3C,F).

When considering high MW aggregate formation, accumulating evidence is supporting a major role of the oligomerization steps in the final outcome. In vitro studies using the small 91-residue N-terminal domain of *Escherichia coli* HypF (HypF-N), a model frequently used for polymerization analyses, have highlighted the capacity of monomers to form distinct oligomeric species that ultimately impact the shape, size, function, and toxicity of the final aggregates [46]. Additionally, molecular modeling studies have helped predict the conformational landscape of oligomers formed from a set of 18 chains of 8-mer polyvaline peptides and the impact on the final aggregate species [47].

Cleavage of MAVS by the NS3/4A protease and consequent detachment from the intracellular membranes [18,48] resulted in a marked decrease in the levels of oligomeric species associated with an impaired antiviral response (Figure 4A). In contrast to oligomers, the levels of high MW aggregates remained unchanged. Rather, in the presence of NS3/4A, the size of aggregates was significantly decreased as revealed by their faster migration compared to aggregates detected in the absence of NS3/4A (Figure 4B). Although these observations suggest that MAVS anchoring to the membrane is essential for the oligomerization, an alternative model would be that cytoplasmic MAVS oligomerizes at a faster rate compared to MAVS localized in intracellular membranes, therefore precluding their detection by immunoblot. This latter hypothesis is supported by previous reports showing that the kinetics of protein aggregation depend on the environment surrounding the oligomers. For instance, the kinetics of p53 and superoxide dismutase 1 (SOD1) aggregation are influenced by the concentration of Zn^2+^ [49,50]. Interestingly, an increased SOD1 polymerization rate is associated with the production of smaller sized aggregates with disorganized amorphous shapes in comparison to the high MW, organized aggregates that assemble into fibril structures [50,51]. It is tempting to hypothesize that a similar process (i.e., faster oligomerization of cytoplasmic MAVS) would explain the formation of aggregates of reduced size compared to MAVS anchored to the membranes. These reduced-size aggregates failed to efficiently induce IFIT1 expression, suggesting that they adopt a structure distinct from the active high MW aggregates. Altogether, these data point to a model in which the formation of MAVS functional high MW aggregates is preceded by appropriate oligomerization that is driven by localization into intracellular membranes. Additional dynamic studies will be required to fully address this model.

Further understanding of how MAVS localization in membranes impacts the formation of oligomers, and as a consequence appropriate aggregation, will require additional structural information encompassing the transmembrane domain, which is not currently available. Based on structural analyses of the RIG-I/MAVS complex, it is accepted that the CARD-CARD interactions act as the central nucleator of the MAVS aggregation process [20,21]. Confirming previous reports, we found that constitutively active RIG-I induces high MW aggregates (Figure 2B). We also report that active RIG-I is sufficient to induce the formation of MAVS oligomers (Figure 1C and Figure 2C). Whether SeV- and RIG-I-induced low MW oligomers and high MW aggregates are identical remains to be addressed. Indeed, sensing of SeV upstream of MAVS involves not only RIG-I but also MDA5. Constitutively active MDA5 was also reported to be sufficient to induce high MW aggregates of MAVS [22]. At this point, it is difficult to appreciate whether RIG-I and MDA5 cooperate to induce specific oligomeric and ultimately aggregate species. Delving more deeply into the physical properties of MAVS oligomers and aggregates triggered by each PRR would benefit from novel techniques allowing structural analysis of the polymers anchored into membranes, such as bacterial expression of transmembrane proteins in the presence of nanodiscs mimicking the lipid bilayer [52,53].

## Abbreviations

The following abbreviations are used in this manuscript:

BME	β-mercaptoethanol
SEC	size-exclusion chromatography
CARD	caspase recruitment domain
FRET	fluorescence resonance energy transfer
HCV	hepatitis C virus
HypF	[NiFe]-hydrogenase maturation factor
IFIT1	interferon-induced protein with tetratricopeptide repeats 1
IFN	interferon
IRF	interferon regulatory factors
ISG	IFN-stimulated gene
MAM	mitochondrial-associated endoplasmic reticulum membrane
MAVS	mitochondrial antiviral signaling protein
MDA5	melanoma differentiation-associated protein 5
MW	molecular weight
NF-κB	nuclear factor kappa-light-chain-enhancer of activated B cells
P53	cellular tumor antigen p53
PRR	pathogen recognition receptor
RIG-I	retinoic acid-inducible gene I protein
SDD-AGE	semi-denaturing detergent agarose gel electrophoresis
SDS-PAGE	sodium dodecyl sulfate-polyacrylamide gel electrophoresis
SOD1	superoxide dismutase [Cu-Zn]
SeV	Sendai virus
TRAF3	TNF receptor-associated factor 3
TRAF6	TNF receptor-associated factor 6
WCE	whole cell extract
